# Biocompatibility Study of Electrospun Nanocomposite Membranes Based on Chitosan/Polyvinyl Alcohol/Oxidized Carbon Nano-Onions

**DOI:** 10.3390/molecules26164753

**Published:** 2021-08-06

**Authors:** Jorge Iván Castro, Manuel N. Chaur, Carlos Humberto Valencia Llano, Mayra Eliana Valencia Zapata, José Herminsul Mina Hernandez, Carlos David Grande-Tovar

**Affiliations:** 1 Grupo de Investigación SIMERQO, Departamento de Química, Universidad del Valle, Calle 13 No. 100-00, 76001 Cali, Colombia; jorge.castro@correounivalle.edu.co (J.I.C.); manuel.chaur@correounivalle.edu.co (M.N.C.); 2 Grupo Biomateriales Dentales, Escuela de Odontología, Universidad del Valle, Calle 4B No. 36-00, 76001 Cali, Colombia; carlos.humberto.valencia@correounivalle.edu.co; 3 Grupo de Materiales Compuestos, Escuela de Ingeniería de Materiales, Facultad de Ingeniería, Universidad del Valle, Calle 13 No. 100-00, 760032 Santiago de Cali, Colombia; valencia.mayra@correounivalle.edu.co (M.E.V.Z.); jose.mina@correounivalle.edu.co (J.H.M.H.); 4 Grupo de Investigación de Fotoquímica y Fotobiología, Facultad de Ciencias, Universidad del Atlántico, Carrera 30 Número 8-49, 081008 Puerto Colombia, Colombia

**Keywords:** biomedical applications, electrospinning, nanofibrous, polyvinyl alcohol, subdermal implantation, tissue engineering

## Abstract

In recent decades, the number of patients requiring biocompatible and resistant implants that differ from conventional alternatives dramatically increased. Among the most promising are the nanocomposites of biopolymers and nanomaterials, which pretend to combine the biocompatibility of biopolymers with the resistance of nanomaterials. However, few studies have focused on the in vivo study of the biocompatibility of these materials. The electrospinning process is a technique that produces continuous fibers through the action of an electric field imposed on a polymer solution. However, to date, there are no reports of chitosan (CS) and polyvinyl alcohol (PVA) electrospinning with carbon nano-onions (CNO) for in vivo implantations, which could generate a resistant and biocompatible material. In this work, we describe the synthesis by the electrospinning method of four different nanofibrous membranes of chitosan (CS)/(PVA)/oxidized carbon nano-onions (ox-CNO) and the subdermal implantations after 90 days in Wistar rats. The results of the morphology studies demonstrated that the electrospun nanofibers were continuous with narrow diameters (between 102.1 nm ± 12.9 nm and 147.8 nm ± 29.4 nm). The CS amount added was critical for the diameters used and the successful electrospinning procedure, while the ox-CNO amount did not affect the process. The crystallinity index was increased with the ox-CNO introduction (from 0.85% to 12.5%), demonstrating the reinforcing effect of the nanomaterial. Thermal degradation analysis also exhibited reinforcement effects according to the DSC and TGA analysis, with the higher ox-CNO content. The biocompatibility of the nanofibers was comparable with the porcine collagen, as evidenced by the subdermal implantations in biological models. In summary, all the nanofibers were reabsorbed without a severe immune response, indicating the usefulness of the electrospun nanocomposites in biomedical applications.

## 1. Introduction

Pathologies affecting hundreds of patients worldwide, including traumas, infections, neoplasm, and failed arthroplasty, remain a serious health issue needing solutions [1]. In general, these patients undergo invasive surgeries that can eventually have unwanted effects and affect the quality of life, resulting in at least four million operations annually [1,2,3]. The primary function of the scaffold is temporarily to imitate the extracellular matrix for cell adhesion and migration [4].

Biopolymers are the preferred materials to produce scaffolds due to their adhesion and biocompatibility advantages [5]. However, the main drawbacks of biopolymers are their low mechanical strength and poor water vapor barrier, affecting new scaffold synthesis. For this reason, it is necessary to incorporate nanofillers or biocompatible synthetic polymers to produce nanocomposites with a set of improved properties such as mechanical strength and high water vapor barrier [6,7]. Biocompatible synthetic polymers are cheaper than biopolymers. Synthetic polymers allow chemical functionalization to improve the mechanical and thermal properties, hydrophilicity, cell bonding, and biodegradability [8,9]. The most used synthetic polymers for scaffold synthesis are poly(glycolic acid) (PGA), poly(lactic-co-glycolic) acid (PLGA), poly(caprolactone) (PCL), poly(l-lactic acid) (PLLA), and poly(vinyl alcohol) (PVA) [10,11]. This latter polymer is a water-soluble, biocompatible, non-toxic, and biodegradable polymer attractive for medical applications due to its better fiber-forming ability [12].

The properties of chitosan (CS) have made it a choice for many researchers as a component for the development of bioactive materials [13]. Chitosan has priceless biological properties due to its unique properties such as high hydrophilicity, biocompatibility, affinity to proteins, intrinsic bacterial and fungal inhibition capacity, suitable for forming hydrogels, films, microspheres, and nanofibers [13,14,15]. However, like many other polysaccharides, CS has the drawback of having low solubility, poor mechanical properties, and poor stability in acidic media. Due to its low solubility, the CS is hardly electrospinnable [16]. Once solubilized, -NH_2_ groups are protonated to -NH_3_^+^, becoming a cationic polyelectrolyte that under an electric field generates repulsion between the positively charged groups [17]. To solve this problem, different researchers mix CS with an easily electrospun polymer such as polyethylene oxide [18], PVA [19,20], among others. Most nanofibers of CS are prepared with PVA due to its favorable interaction at the molecular level [21,22].

Since the discovery of electrospinning, applications have increased rapidly over the past decades because it is a cost-effective and straightforward technique involving applying an electric field to liquid polymers [23,24,25,26]. Generally, upon different conditions and polymer variations, the diameters produced by electrospinning vary broadly (from 5 to 500 nm) [27]. Furthermore, the nanofibers produced by the electrospinning method have shown exciting properties concerning the area and the volume supplied in a specific area and high porosity with a tiny pore size [28]. Those properties make them applicable in the textile industry and the manufacture of filters [29,30]. On the other hand, the fibers possess considerable static charges, which allow manipulation in three-dimensional structures during their deposition [31,32]. Besides the above-described properties, electrospun nanofibers have been applied in tissue scaffolds, drug release, and wound dressings based on biocompatibility and tailor-designed properties [33].

The favorable characteristics shown by carbon nanomaterials such as carbon nano onions (CNO) make them suitable for regenerative medicine, drug delivery [34], imaging [35], and sensing [36,37]. The primary synthetic method to produce CNO uses thermal annealing of detonation nanodiamonds at high temperature, vacuum, and inert atmosphere. This method produces CNO with 5–6 nm in diameter and 6–8 graphitic shells [38,39]. CNOs have been used in biomedical applications by several research groups. For example, Pakhira et al. improved the transport across the blood-brain barrier of an anti-Alzheimer’s drug-using water-dispersible CNOs. The researchers showed that the drug entered the brain without any affectation [34]. Lettieri et al. functionalized *p*-CNO (pristine carbon nano onions) with boron dipyrromethene molecules, resulting in fluorescent CNO (fluo-CNOs) with high dispersibility in an aqueous medium with low toxicity and easy uptake by human breast cancer cells [35]. Bartolome et al. fabricated a sensor of DNA and CNOs to detect DNA sequences of the human papillomavirus genome [36].

Recently, we reported the synthesis by the drop-casting methodology of film nanocomposites based on chitosan/poly(vinyl alcohol)/ox-CNO mixture [40] and chitosan/poly(vinyl alcohol)/CS-g-CNO covalently grafted [41]. These studies demonstrated a higher biosorption and lower remaining material without any aggressive immune system response. Besides, CNO grafted with chitosan improves the biocompatibility and reabsorption of the film’s simulated tissue regeneration. However, films produced by the described methodology did not present porosity, which is a fundamental requirement for cell adhesion and renewal. With that in mind, this work describes the synthesis and physical-chemical characterization of four formulations of nanofibers prepared by the electrospinning technique based on CS/PVA/ox-CNO and their biocompatibility study in Wistar rats using subdermal implantations. With the investigation, we pretend to understand if the nanofibers produce an aggressive immune response or toxicity by analyzing the implantation histology data. The investigation results will contribute to the biomedical field knowledge and postulate the nanofibers as cell-adhesion and proliferation candidate scaffolds for medicine applications.

## 2. Results and Discussion

### 2.1. Nanocomposite Characterization

For the CS/PVA/ox-CNO electrospun fibers used in this study, it can be expected that the interaction between the components will be found between the hydroxyl groups of both the PVA and the chitosan and the surface of the ox-CNO rich in oxygenated groups such as -OH, -COOH and even epoxy, previously confirmed by our group through analysis by RAMAN, FT-IR, XRD, and TGA spectroscopy [40]. Similarly, the interaction between the hydroxyl groups of CS, PVA, and the oxygenated groups of graphene oxide has been reported [42]. Both nitrogen and oxygen atoms have lone electrons that can electrostatically bond a proton through a pair of electrons they share in forming H-bonds. Due to the stronger attraction of the lone pair of electrons to the nucleus by oxygen than a nitrogen atom, nitrogen atoms would have a greater tendency to donate the lone pair of electrons to form an H-bond with the hydroxyl groups of PVA and ox-CNO [1,2].

During the implantation experiments, the pH of the subdermal zone of the biomodels can be slightly acidic as a result of the inflammation process. CS could be partially protonated (pKa~6.5) [43], which would facilitate its electrostatic interaction with the covalent network of nano-onions that tends to have a negative charge due to the presence of π electrons, as has also been observed before in graphene oxide [44,45]. It is also possible that van der Waals type interactions (London dispersion forces, charge-dipole, dipole-dipole, dipole-induced dipole) and π interactions (polar-π, charge-π, lone pair-π or n-π *, dipole-π) are generated. The non-covalent interactions are possible due to a large number of carbon-hydrogen bonds present in both chitosan and PVA, free pairs present in the oxygen atoms of PVA and CS, as well as the atoms of nitrogen of the CS and, of course, the dipoles formed, which can interact with the ox-CNOs and their oxygenated functional groups on the surface [46].

Another essential aspect to highlight is that incorporating PVA into CS improves mechanical strength and hydrophilicity, thus improving the interaction with the body environment through H-bonds and electrostatic interactions (salt bridge) with charged groups from other molecules in cells [47]. The characterization of the nanocomposites and the component’s interactions are presented below:

#### 2.1.1. Fourier-Transform Infrared Spectroscopy (FT-IR)

**Figure 1:**
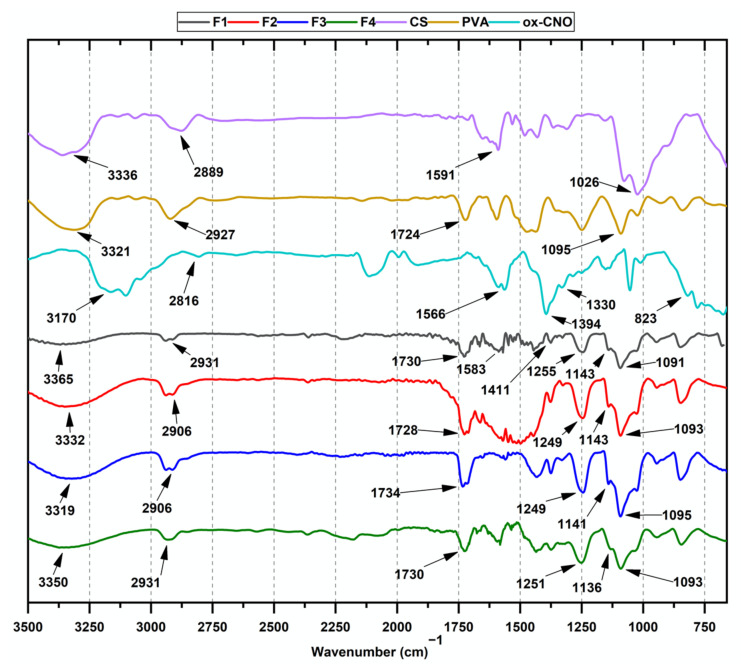
Attenuated total reflectance Fourier-transform infrared spectroscopy (ATR-FT-IR) of electrospun CS/PVA/ox-CNO composite nanofibrous membranes: F1 (CS:PVA:ox-CNO 8:92:0); F2 (CS:PVA:ox-CNO 8:91:1); F3 (CS:PVA:ox-CNO 3.5:95.5:1); F4 (CS:PVA:ox-CNO 5.4:92.6:2).

Figure 1 shows the FT-IR spectrum of the nanocomposite fibers. The bands at 3291 and 2914 cm^−1,^ which are assigned to -OH and -CH_2_ stretching from PVA, are observed for F1 (CS:PVA:ox-CNO 8:92:0). The bands at 1730, 1583, and 1255 cm^−1^ correspond to the C=O of amides, N-H bending of NH_2,_ and CH_2_ groups from CS. The band at 1411 cm^−1^ corresponds to oscillations of OH and CH groups [42]. Likewise, nanofibers F2 (CS:PVA:ox-CNO 8:91:1); F3 (CS:PVA:ox-CNO 3.5:95.5:1), and F4 (CS:PVA:ox-CNO 5.4:92.6:2) exhibit a band at 3365 cm^−1^ of -OH group shifted to a higher wavenumber compared to the original CS: PVA composites due to the strong interaction between CS/PVA/ox-CNO discussed above.

Moreover, the acetamido asymmetric stretching bands were observed at 1560 and 1091 cm^−1^ for all the nanofibers. The band at 1095 cm^−1^ of the different nanofibers is due to the vibration of C-O bonds in PVA. However, the band’s intensity decreases as the amount of CS increases, as seen for F4. The band at 1260 cm^−1^ is related to C-O stretching vibrations. Two bands at 1377 and 1432 cm^−1^ are assigned to CH-OH and CH_2_ symmetric bending vibrations of PVA [48].

On the other hand, the electrospun F2, F3, and F4 presented bands at 1730 cm^−1^ related to the carbonyl group of the ox-CNO [49], overlapped with the characteristic bands of the amide groups. Furthermore, the presence of the bands C-O-H at 960 cm^−1^, stretching vibration C-O at 1249 cm^−1,^ and the deformation in the plane of O-H at 1330 cm^−1^ confirmed the ox-CNO incorporation in the nanofibers. Furthermore, the C-N band between 1374 and 1600 cm^−1^ for F2 shows a widening, probably due to the loss of hydrogen bonds between CS and PVA and the new hydrogen bonds formed with the ox-CNO. Finally, the bands at 1647, 1558, and 1327 cm^−1^ are related to C=O from amides, N-H bending of NH_2_, and CH_2_ from CS in the nanocomposite. The FT-IR results confirmed functional groups of CS, ox-CNO, and PVA into the nanofibers and their chemical interaction.

#### 2.1.2. X-ray Diffraction (XRD)

**Figure 2:**
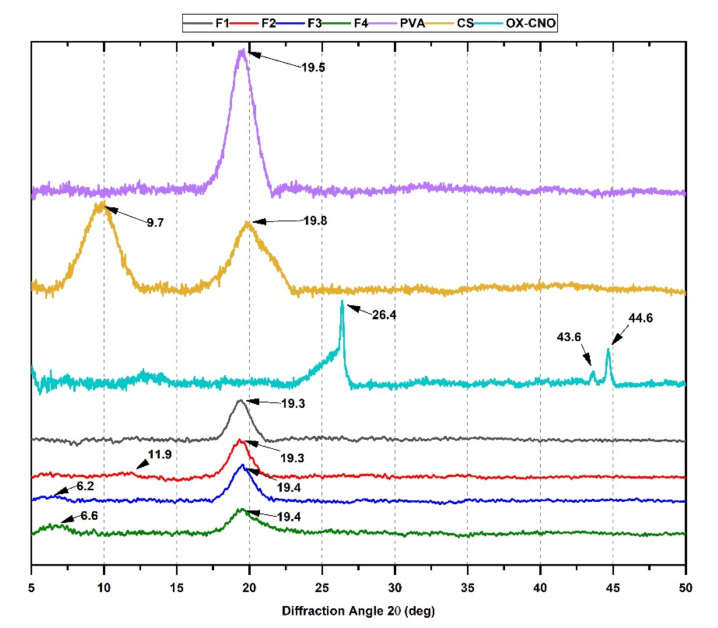
X-ray diffraction (XRD) analysis of CS/PVA/ox-CNO formulations: F1 (CS:PVA:ox-CNO 8:92:0); F2 (CS:PVA:ox-CNO 8:91:1); F3 (CS:PVA:ox-CNO 3.5:95.5:1); F4 (CS:PVA:ox-CNO 5.4:92.6:2); CS, PVA, and ox-CNO.

The XRD patterns of the CS/PVA/ox-CNO nanofibers are shown in Figure 2. One peak with high intensity at 2θ = 19.5° is due to the high number of hydrogen bonds in the semicrystalline structure of PVA [50]. The diffraction of pure CS showed two typical peaks at 2θ = 9.7° (020) and 2θ = 19.8° (110) associated with the crystalline structure [51,52]. For ox-CNO, there are three peaks at 2θ = 26.4°, 2θ = 43.6°, and 2θ = 44.6°, corresponding to the diffraction planes (002), (111), and (100), respectively [51]. On the other hand, the nanofibers exhibited two peaks at 2θ = 6.2° and 2θ = 19.3°, corresponding to the (020) plane of CS and (101) planes related to the PVA crystals. The shifting of the peaks demonstrates a strong interaction between the nanocomposite components, a result previously observed for PVA films and nanofibers [53].

Moreover, to understand better the changes in the crystallinity for the nanocomposites, we studied the crystallinity index (Xc) as reported in Table 1. The methodology is an adaptation from the original purpose for starch crystallinity determination [54].

The crystalline structure is affected when CS, PVA, and ox-CNO are mixed, as shown by the crystallinity indexes in Table 1. The shallow crystallinity values are the result of the electrospinning process. It is well known that electrospinning delays the crystallization in nanocomposites affecting the development of the crystalline microstructure [53].

Obtaining fibers by electrospinning generates high elongation rates quickly, while the fibers solidify, which prevents the crystallization of the samples [27,53]. As a result, the Deborah number (the ratio between the time of the phenomenon with the time of the experiment) is more significant than one, meaning that the time scale is shorter than when the phenomenon occurs [55]. In addition, the solvent evaporates in seconds, while the crystallization takes days. Finally, the nanofiber morphologies are affected by the delay in forming the crystalline microstructure [48].

On the other hand, ox-CNO incorporation in formulations (F2, F3, and F4) caused an increase in the crystallinity index. This effect has been reported using other carbon materials such as nanocrystals, carbon nanotubes, graphene oxide [42,56,57]. The enhanced Xc can be attributed to the higher crystallinity of the ox-CNO, corroborated in the diffractogram with the presence of narrower and sharper peaks for these, in comparison to the broadened peaks exhibited by the individual polymers, added to a high surface area which facilitates an increased interaction between the oxygenated surface groups of ox-CNO and the polymer matrix (CS and PVA) provided [58].

#### 2.1.3. Thermal Analysis

We determined the thermogravimetric analysis (TGA) and the degradation profile based on the degree of functionalization in the nanofibers. The thermal decomposition of CS (Figure S1A) and PVA (Figure S1B) are available in the Supplementary Material. The deterioration of CS undergoes three phases. First, a weight loss between 40–140 °C is related to volatiles removal, such as free water. The second phase starts at 220 °C, corresponding to structural water loss. Finally, the third phase begins at 320 °C related to the degradation of glycosidic bonds of chitosan [59].

For PVA (Figure S1B), three stages of degradation are evident. The first stage of weight loss began at 50–170 °C that results from the water ebullition and the loss of the most volatile molecules. The second weight loss begins at 230 °C, occasioned by PVA side chains decomposition. Finally, the third stage starts at 400 °C, corresponding to further degradation of polyene residues [59].

**Figure 3:**
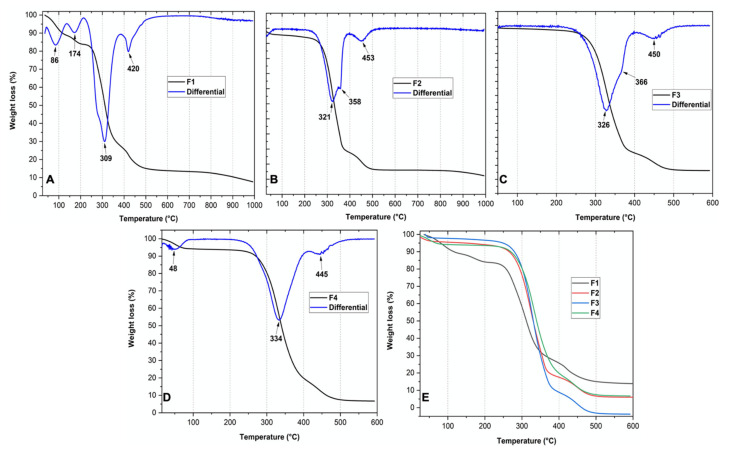
TGA curves of the nanofibers. Formulations: F1, (**A**) (CS:PVA:ox-CNO 8:92:0); F2, (**B**) (CS:PVA:ox-CNO 8:91:1); F3, (**C**) (CS:PVA:ox-CNO 3.5:95.5:1); F4, (**D**) (CS:PVA:ox-CNO 5.4:92.6:2); (**E**) all the formulations.

For the nanofibers, F1 and F2 (Figure 3A,B) with a higher content of CS than F3 and F4 (Figure 3C,D), the initial weight loss between 50–175 °C of CS/PVA/ox-CNO is attributed to the water loss; like CS, F1 also shows the second stage of thermal degradation at 220 °C, presumably due to the higher CS content. F2 does not offer this behavior because of the incorporation of the ox-CNO. The increase in the PVA content (F3 and F4) shows a similar thermal degradation as PVA (Figure S2, Supplementary Material) with less water retained and less weight lost before 300 °C.

Increasing CS between F3 and F4 and decreasing the amount of PVA shows a higher amount of volatile content between 50 and 270 °C with a higher weight loss for F4. Furthermore, increasing CS content also shows more weight retention after 350 °C because of more inorganic content in CS (like F1). On the other hand, the reinforcement effect of ox-CNO incorporation can be observed in comparing the thermal degradation profiles of F2 and F4. In that case, F2 has more CS content than F4 (8 wt.% vs. 5.4 wt.%), but F4 has more ox-CNO range than F2 (2.0 wt.% vs. 1.0 wt.%). Thus, for F4, more weight remains between 350–400 °C, due to the ox-CNO incorporation. Above that temperature, the degradation is attributed to the decomposition of carbon nano-onions. Finally, between F1 and F2, the reduction of PVA in 1.0 wt.% and incorporation of 1.0 wt.% of ox-CNO introduced a reinforcement mainly observed above 100 °C where F2 retained more weight until 350 °C.

**Figure 4:**
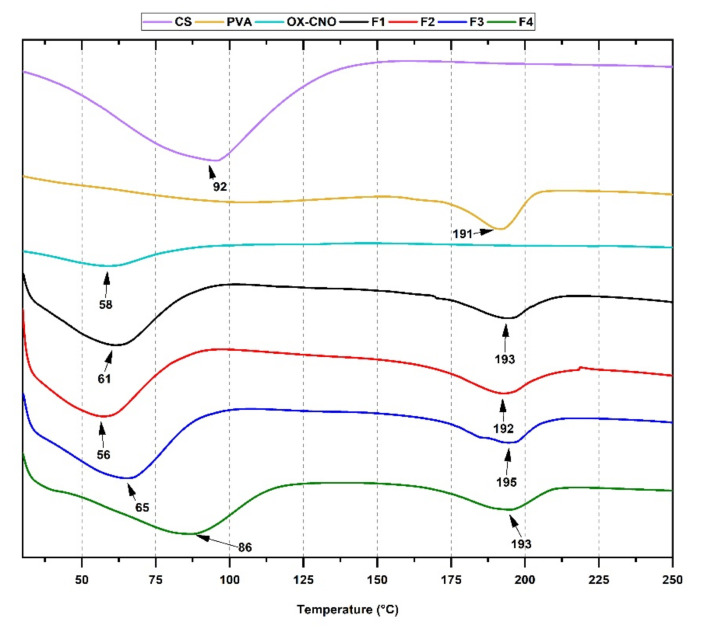
DSC curves of the nanofibers. Formulations: F1 (CS:PVA:ox-CNO 8:92:0); F2 (CS:PVA:ox-CNO 8:91:1); F3 (CS:PVA:ox-CNO 3.5:95.5:1); F4 (CS:PVA:ox-CNO 5.4:92.6:2).

The DSC curves analysis allows us to understand the phase transitions of materials (Figure 4). However, the glass transition (Tg) and the melting temperature (Tm) are influenced by the experimental conditions and sample preparation, and the addition of additives and processing methodologies. A broad endothermic peak was observed in the CS thermogram starting from 25 °C and ending at 150 °C. This transition temperature is due to water molecules trapped in the CS structure in three different forms. The first form corresponds to freezing water being released at about −5 °C. The second form corresponds to structural water evaporation in a range of 10 °C and 100 °C, and the third form fits to free bound water release at about 150 °C [60].

Furthermore, due to the intramolecular bonds, chitosan is not considered a thermoplastic. Therefore the degradation point occurs before the melting point for the crystal structure [61]. In the PVA thermogram, the endothermic peak at 191 °C indicated the Tm for PVA. Usually, the Processing of PVA segments causes a rapid dehydration chain scission [62]. A broad endothermic peak was observed in the ox-CNO thermogram starting from 25 °C and ending at 98 °C, probably related to the water entrapped within the crystalline structure.

In the overall thermogram for nanofibers, displacements of endothermic peaks were observed at lower temperatures, characteristic of compounds containing CS and PVA [63]. The decrease in the temperatures may be due to the different orientations taken by the chains when interacting with an electric field for PVA and the other forms of intrinsic water when subjected to this external vector for CS [60]. The displacements of the endothermic peaks agree with the crystalline structure changes discussed in the XRD section due to the electrospinning processing. Besides, the broad peak at the beginning of the thermogram for nanofibers could also be related to some remanent acetic acid solvent (the boiling point of acetic acid, 110 °C). Particularly for F4, the increase in the endothermic peak is shifted to a higher temperature, probably due to the crystallinity reinforcement with the increased ox-CNO content, providing significant material stability by forming hydrogen bonds intramolecularly with PVA and CS [48].

#### 2.1.4. Scanning Electron Microscopy (SEM)

The morphology and diameter of electrospun fibers are affected by several variables, such as nanomaterial forming dispersion, material processing, and environmental conditions. Between those parameters, the control of the solution parameters (concentration, viscosity, conductivity, molecular weight, and surface tension) is one of the most effective ways to control the morphology and diameter of the fibers [64,65].

In this study, we did not carry out measurements of the surface charge of the nanofibers because the functionalization is explained directly from the characterization of XRD, FT-IR, SEM, and TGA and that the main objective of the work was the analysis of the biocompatibility of the In vivo nanocomposites in biomodels. Charge density and aggregation of nanomaterials have been shown to influence their in vitro cytotoxicity [66,67]. Although the ζ-potential is a determining factor in the colloidal stability of nanomaterials, it does not necessarily represent the particular state in different environments [44]. When implanting the nanofibers, the nano-onions are not directly in a colloidal state but are distributed in the polymeric matrix of CS and PVA with the different non-covalent, and electrostatic interactions previously explained. Therefore, we decided to analyze the possible aggregation or coagulation through morphology analysis by SEM, which allowed us to directly see the formation of nanofibers and at very high percentages of CS beads. However, it is possible to hypothesize that the main interactions between the components of the CS/PVA/ox-CNO nanocomposites have occurred thanks to the oxygenated groups on the surface of ox-CNO and the hydroxyl and amino groups in CS and PVA.

Additionally, ox-CNOs are multilayered carbon structures with multiple sp^2^ carbons and π electrons, even though on the surface, functionalization by oxidation reactions affects this covalent network by generating sp^3^ carbons. Therefore, the surface charge of the carbon nano-onions could be slightly negative, as has been shown for graphene oxide and for reduced graphene, which allows it to interact electrostatically with CS, which could be partially positively charged by its pKa~6.5 [43], what has been previously shown for graphene oxide and graphene [44,45]. This negative surface charge allows CS and graphene oxide nanocomposites to interact with viruses and phosphatidylcholine lipids present on the outer membrane of many cells. The PVA would contribute to the partial negative charge, which is why it would also help the interaction with the partially positive CS and perhaps, interact with cells, which benefit the reabsorption process of the membranes.

**Figure 5:**
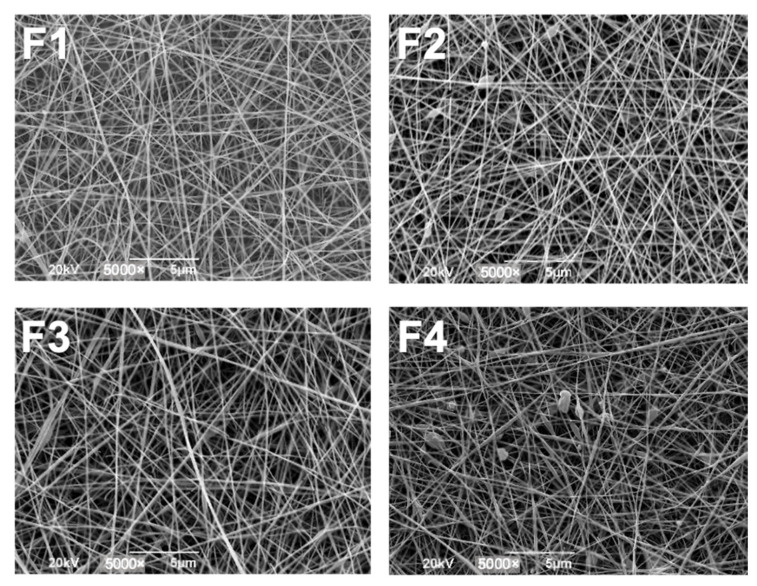
SEM images of electrospun CS/PVA/ox-CNO composite nanofibrous membranes. Formulations: (**F1**) (CS:PVA:ox-CNO 8:92:0); (**F2**) (CS:PVA:ox-CNO 8:91:1); (**F3**) (CS:PVA:ox-CNO 3.5:95.5:1); (**F4**) (CS:PVA:ox-CNO 5.4:92.6:2). For all the experiments, the voltage used was 20 kV, and its images at 5000×.

In Figure 5, the production of more beads could be observed when the concentration of ox-CNO was approximately 2.0 wt.% (F4). This change in morphology is based on the ox-CNO addition, decreasing the solution’s viscosity [68]. The carboxylate groups of carbon nano-onions contain charges that generate repulsion with PVA hydroxyl groups when subjected to an electric field, affecting the formation of continuous fibers. In addition, when the percentage of CS was varied, keeping the ox-CNO amount constant (F2 and F3), a change in the diameter of the fiber was observed, accompanied by a decrease in the pearlescent bodies in the nanomaterial (Table 2). The polyelectrolyte character of CS increases the conductivity of the solution, reducing self-repulsion due to excess charges and achieving an increase in the size of the fiber [53,65]. However, as the percentage of the CS increases above 95%, the solution was more viscous, and the formation of fibers does not occur.

The nanofibers diameters are presented in Table 2, and the SEM images with the diameters measured are shown in the Supplementary Material (Figure S2). Formulation F4 showed the lowest diameter (102.1 nm ± 12.9 nm) and F1 the highest (147.8 nm ± 29.4 nm). The average diameter is reduced as the content of CS increases, while the addition of ox-CNO has almost no effect on the diameter. Electrical field instability induction might be the most plausible cause of the increased diameters with CS increasing. CS limits the production of fibers due to its poly electric nature in an acidic medium, increasing the surface charges in the jet, provoking a decrease in the size of the fibers. This phenomenon has been observed in previous works on CS and PVA electrospun fibers [53,69].

#### 2.1.5. Biomodel Tests In Vivo

CS scaffolds provide interconnectivity and neo-vascularization through implantation experiments [70]. It has been proved that chitosan behaves as an extracellular matrix and provides a microenvironment for cell proliferation, which is of great interest for biomedical applications like wound healing and regenerative medicine [71,72]. Chitosan initiates fibroblast cells’ proliferation, stimulates collagen synthesis, supports tissue growth and cell differentiation due to the resemblance with glycosaminoglycans distributed throughout the human body [71,72,73,74,75,76,77].

**Figure 6:**
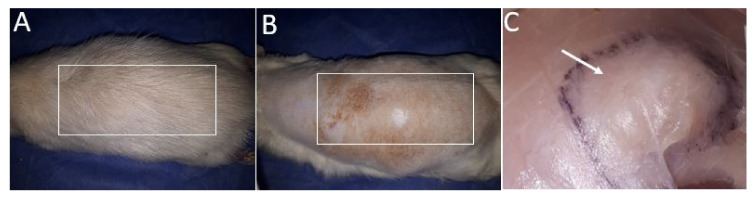
Implantation zone at the dorsal area in Wistar rats after 30 days of subcutaneous implantation: (**A**) hair recovery, (**B**) absence of injuries and infections, and (**C**) internal surface of the skin where the implanted samples are encapsulated by scar tissue. Rectangle: Area of implantation. Arrow: encapsulation zone.

Histology assays of the nanocomposite fibers were assessed after 30 and 90 days of implantation, following the recommendations of the ISO 10996 (UNE 10996) standard. The biomodels were euthanized 30 and 90 days after implantation. Macroscopic observations found normal hair formation (Figure 6A). Completely healthy skin is observed when trichotomy was performed (Figure 6B). On the inner surface of the skin, the implantation areas of the nanofibers were identified after 30 and 90 days without the presence of exudates, signs of degradation, severe inflammation, redness, presence of purulent exudate, or bad smell. Figure 6C shows an image of an encapsulation zone 30 days after the materials were implanted.

The encapsulation of the inserted material is part of the scar response to an implanted material. It is a strategy that allows the repair of the injured tissues during the surgical intervention, while the body’s cells resolve the situation presented by the presence of the foreign material [78]. The histological image observed in the area adjacent to the implanted material depends on the extent of the surgical lesion and the cells present in the lesion. Some cells have tissue repair functions, while others are responsible for eliminating the material [79].

The materials implanted in this investigation were encapsulated in the subdermal area, while the other tissues showed an appearance of healthy tissue, an indication of the biocompatibility of the materials used. The magnitude of the histologically observable response will depend on the extent of the lesion created and the type of grafted material [78]. The results of what was found 30 and 90 days after implantation of each material are presented below. The histological images correspond to what happened inside the capsule in each area.

**Figure 7:**
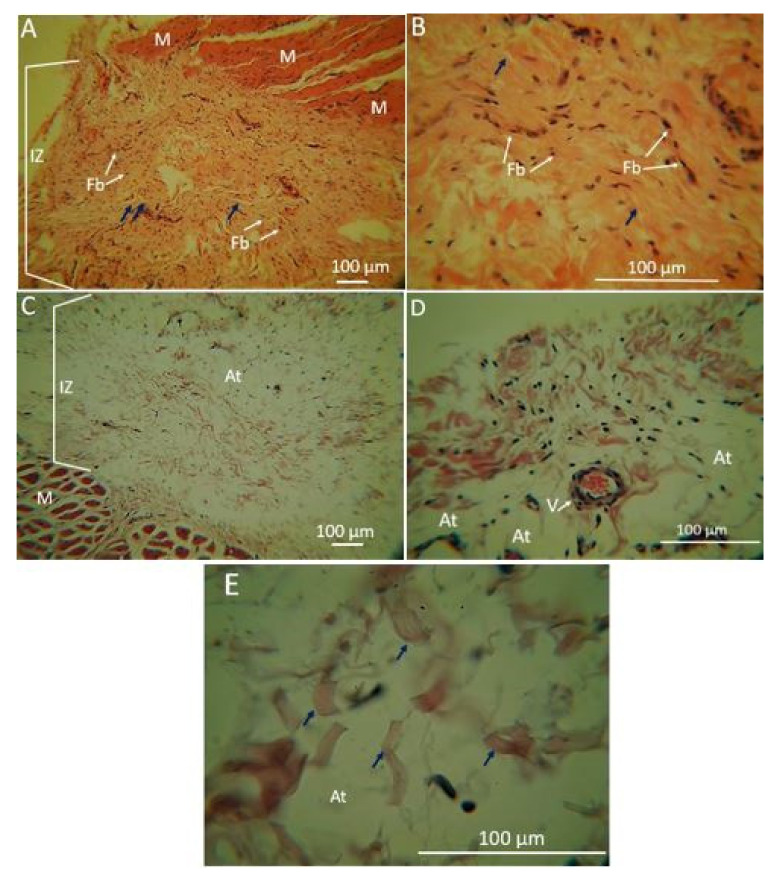
Nanocomposite F1 implanted in the Wistar rat subdermal area at 30 and 90 days. (**A**) at 10× implantation at 30 days. (**B**) at 40× implantation at 30 days. (**C**) at 10× implantation at 90 days. (**D**) at 40× implantation at 90 days. (**E**) at 100×, implantation at 90 days. IZ. Implantation area. M: Muscle. Fb: Fibroblasts. At: Adipose tissue. Blue arrow: polymeric fibers (sample). V: Blood vessel. Hematoxylin and Eosin Technique (H&E).

Nanocomposite F1 after 30 days of implantation presents a prominent inflammatory infiltrate with abundant blood vessels and the presence of the implanted material, as shown in Figure 7. After 90 days (Figure 7C–E), the inflammatory infiltrate persists, and the fibers of the implanted material are visible, although the resorption of the material is evident when comparing the image with Figure 7A,B. The reabsorption reflects the remarkable stability of the CS: PVA (F1) nanocomposite, thanks to the hydrogen bonds that present their chains, as has been previously established [58]. Chitosan is a material that exhibits in vivo phagocytosis and enzymatic activity, dependent on the degree of deacetylation, cross-linking, binding with other polymers, and the synthesis process for the scaffolds [80,81]. In addition, several researchers have demonstrated biocompatibility, the ability to degrade, and the inducing effect for the regeneration of chitosan tissue [71,72,73,74,75,76,77,82,83].

Fibroblasts are cells involved in tissue repair and regeneration in the early stages of scarring [79]. The absence of an inflammatory infiltrate indicates that the inflammatory process is resolving. The two fair events would suggest that a resolution of the situation generated by implantation through tissue repair occurs.

In Figure 7D, a sample with the same formulation is observed at 90 days of implantation. An almost complete scar response is observed with the recovery of the tissue architecture of the implanted area (IZ) with an abundant presence of adipose tissue. Figure 7C corresponds to the exact location at higher magnification, showing a large blood vessel. The histological image is compatible with tissue healing, with the absence of inflammatory infiltrate. After 30 and 90 days, fragments of the implanted material are observed. However, due to the translucency of these fibers, they are more evident when using immersion oil at a magnification of 100× (Figure 7E).

Histological analysis of this sample indicates that the material is stable at three months, presenting low degradability and good biocompatibility, evidenced by tissue repair with the recovery of tissue architecture in the absence of inflammatory infiltrate.

**Figure 8:**
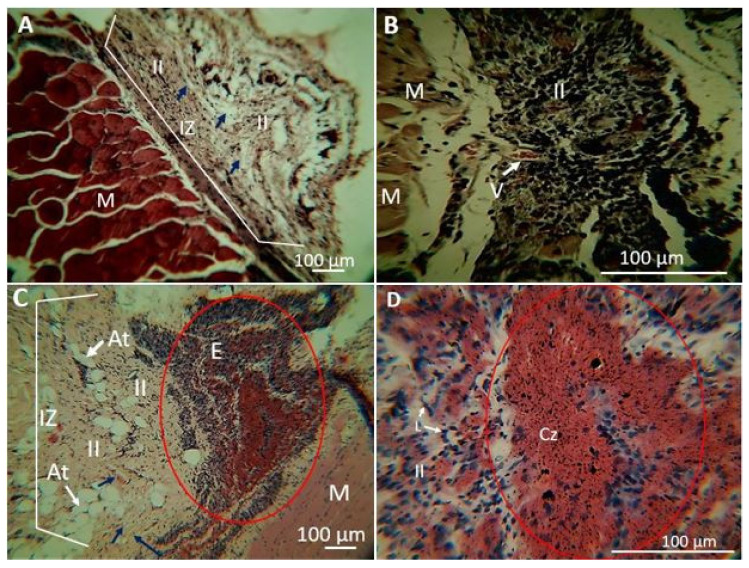
Nanocomposite F2 implanted in the Wistar rat subdermal area at 30 and 90 days. (**A**) at 10× implantation at 30 days. (**B**) at 40× implantation at 30 days. (**C**) at 10× implantation at 90 days. (**D**) at 40× implantation at 90 days. IZ. Implantation area. II: Inflammatory infiltrate. M: Muscle. V: Blood vessel. L: Lymphocytes. Blue arrows: implanted material. Red oval: extravasation zone during sample recovery. E: erythrocytes Cz: coagulation zone. Hematoxylin and Eosin Technique (H&E).

Figure 8 corresponds to formulation F2. At 30 days, slight inflammatory infiltrates of lymphocyte conformation are observed (Figure 8A), with the presence of blood vessels (Figure 8B) and fibers of the material in the process of degradation (blue arrows in Figure 8A). After 90 days of implantation, a decrease is observed in the inflammatory infiltrate and the presence of small portions of the implanted material (blue arrows in Figure 8C), which indicates that the material begins to be reabsorbed very well, demonstrating that the presence of ox-CNO does not affect the biocompatibility process of the material while it reinforces it. Histological analysis indicates that this material is also stable and biocompatible, presenting a slight inflammatory infiltrate of the lymphocytic type that decreases after 90 days. The infiltrate can be attributed to the presence of the implanted material.

**Figure 9:**
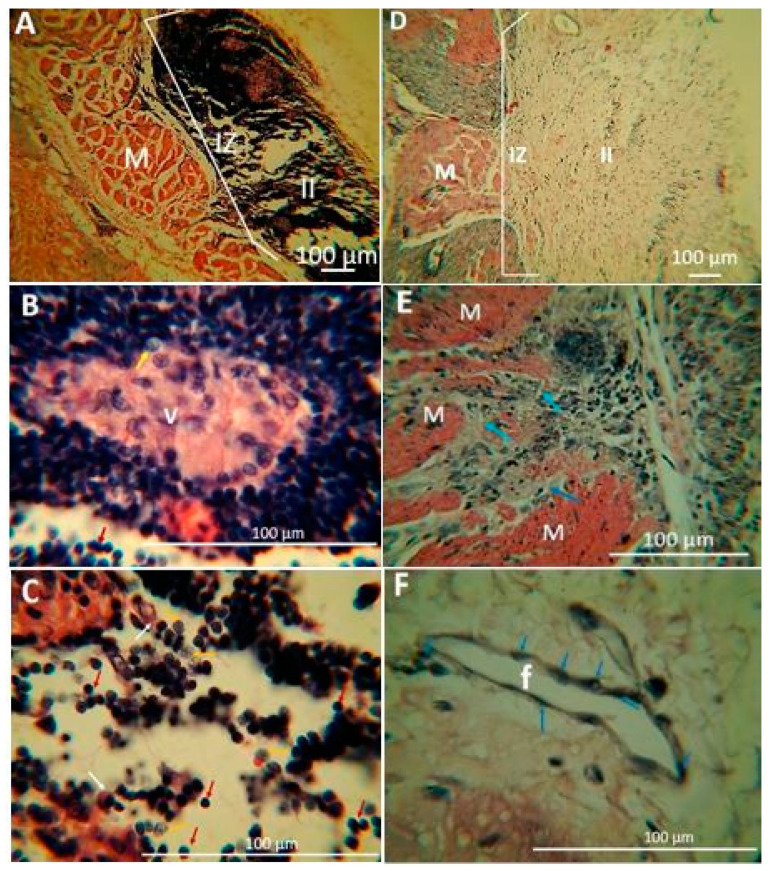
Nanocomposite F3 implanted in the subdermal area of Wistar rats at 30 and 90 days. (**A**–**C**) correspond to the nanocomposites after 30 days of implantation. (**D**–**F**) correspond to the nanocomposites after 90 days of implantation. (**A**) at 10×. (**B**) at 100×. (**C**) at 100×. (**D**) at 10×. (**E**) at 40× and (**F**) at 100×. IZ. Implantation area. M: Muscle. II: Inflammatory infiltrate. V: Blood vessel. Yellow arrows: implanted material. White arrow: Plasmocyte. Blue arrow: Histiocytes. Red arrow: Lymphocyte. f: Fiber. Hematoxylin–Eosin Technique (H&E).

Figure 9 corresponds to the nanocomposite F3 after 30 days of implantation in subdermal tissue. Figure 9A corresponds to the sample with 30 days of implantation. A broad focus of inflammatory infiltrate (II) is observed in the implantation zone (IZ). At 40× magnification (Figure 9B), a large blood vessel can be seen, and abundant lymphohistoplasmacytic inflammatory infiltrates (Figure 9C).

After 90 days, the inflammatory infiltrate is more of the lymphocytic type (Figure 9D), and small residues of the implanted material are still observed (Figure 9E). It is also possible to observe that the material remains encompassed by a histiocytic infiltrate (higher magnification 100×, Figure 9F).

**Figure 10:**
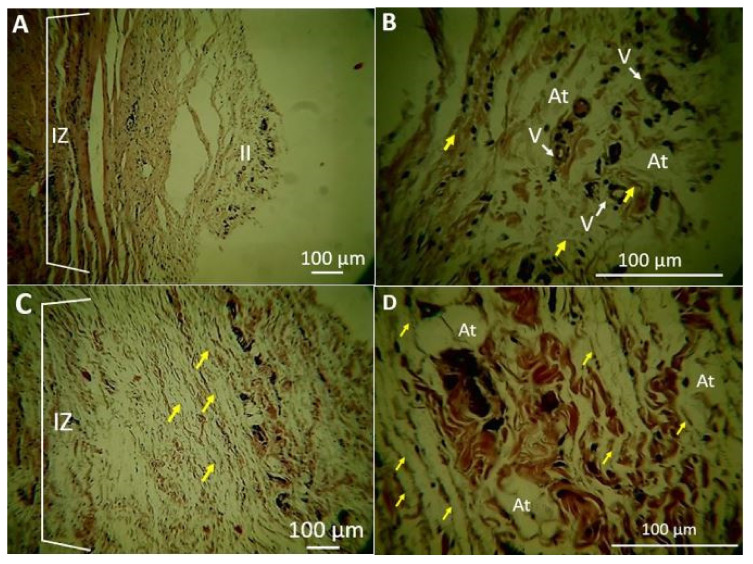
Nanocomposite F4 implanted in the subdermal zone of the Wistar rat at 30 and 90 days. (**A**,**B**) correspond to 30 days, (**C**,**D**) correspond to 90 days. (**A**) at 10×. (**B**) at 40×. (**C**) at 10× and (**D**) at 40×. IZ. Implantation area. II: inflammatory infiltrate V: blood vessel. At: adipose tissue. Yellow arrows: implanted material. Hematoxylin and Eosin Technique (H&E).

Nanocomposite F4 after 30 days shows the presence of an inflammatory infiltrate, and some fibers of the implanted material are also observed (Figure 10).

Histological studies of the nanocomposite F4 at 30 days (Figure 10A) show the presence of fibers from the implanted material in a slight inflammatory lymphocyte-type infiltrate with the presence of blood vessels. At 90 days, the persistence of the implanted material and a small inflammatory lymphocyte type infiltrate at the tissue repair site with adipose tissue were observed, indicating that the material is biocompatible and very stable under physiological conditions during the implantation period.

**Figure 11:**
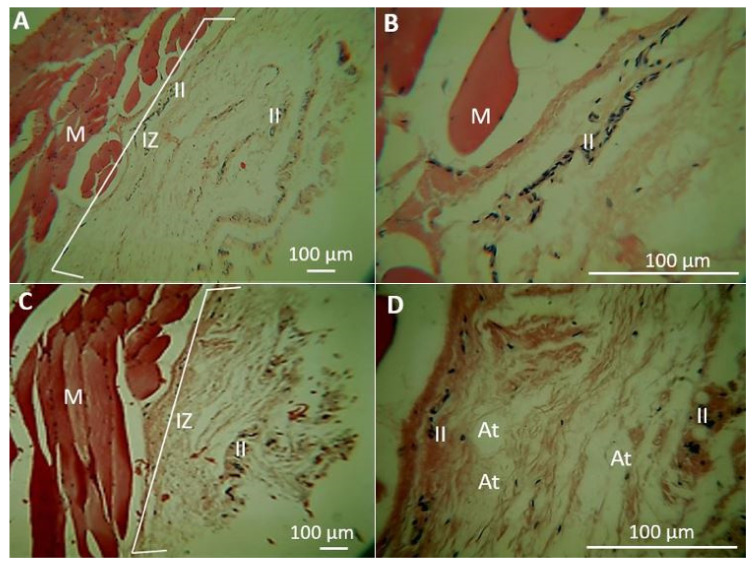
Porcine collagen (Control) implanted in the Wistar rat subdermal area for 30 and 90 days. (**A**,**B**) correspond to implantation at 30 days, (**C**,**D**) to implantation at 90 days. (**A**,**C**) at 10×. (**B**,**D**) at 40×. IZ. Implantation area. II: Inflammatory infiltrate. M: Muscle. At: adipose tissue. Hematoxylin Eosin Technique (H&E).

The control sample corresponds to the collagen sample. At 30 days, there is almost complete reabsorption of the material with some inflammatory cells (Figure 11A,B). Figure 11C corresponds to the implanted control sample at 90 days. The presence of a mild inflammatory infiltrate corresponds to small fragments of the implanted material. The histological image is of a repaired tissue with the establishment of adipose tissue, which is typical of the area of the hypodermis where the material was implanted.

In all the cases studied, fibrous scar tissue surrounding the implanted material was observed, being more noticeable macroscopically in the samples at 30 days than those at 90 days, which is considered a typical healthy healing response observed with most samples implanted materials [78,79].

When studying the implanted areas microscopically, it was observed that within the fibrous scar area, a scarring process was taking place characterized by the presence of repairing cells such as fibroblasts, structures such as blood vessels necessary for the transport of cells and nutrients, as well as for the elimination of waste products, with the presence of a lymphocytic-type inflammatory infiltrate that diminishes over time.

In the repair process, when materials are implanted, if they are not degraded or reabsorbed, they are surrounded by a capsule of collagen fibers. However, this situation should not be considered an indicator of a lack of biocompatibility but rather a slower reabsorption process [84].

Our results do not show that the implanted materials were surrounded by a fibrous capsule, indicating rapid resorption of the material. Figure 9F shows a fragment of one of the implanted polymeric fibers surrounded by cells compatible with histiocytes, which could be the beginning of a fibrous encapsulation process.

F1 and F2 nanocomposites presented a mild lymphocyte-type inflammatory response, while other cells were found in the F3 nanocomposite, such as histiocytes and plasma cells. This situation possibly occurred due to the persistence of more stable fragments (those related to the crystalline regions of the polymers with high non-covalent interactions between them and ox-CNO) of the implanted material that prompted a foreign body reaction of greater intensity since they could not be phagocytosed. Usually, the higher crystalline degree is directly related to lower enzymatic hydrolysis for polysaccharides (i.e., cellulose, starch, chitosan) [85].

When a wound occurs, a healing process is stimulated composed of three phases: inflammation, proliferation, and remodeling aiming to close the wound and recover the tissue either through regeneration with the recovery of the scar architecture or by forming scar tissue [86].

It has been determined that the repair process occurs through an inflammatory response that can be acute or chronic. The acute generally occurs in the first week and does not usually last more than 15 days. In addition, there is an abundant presence of cells of different lineages such as mast cells, lymphocytes, plasma cells, and fibroblasts. On the other hand, chronic inflammation is established after 15 days if a factor predisposes to maintaining the inflammatory response. For instance, an implanted biomaterial is considered a foreign body and is characterized by polymorphonuclear cells such as histiocytes if the injury occurs in connective tissue, and the reaction is confined to the implantation site [87].

The chronic phase evolves towards complete material reabsorption or fibrous encapsulation depending on the characteristics of the material, such as crystallinity and ease of enzymatic degradation [88,89].

The presence of lymphocytes in the implantation sites is associated with recognition and cellular communication, especially with macrophages in the chronic phase [86]. A predominantly lymphocytic cell infiltrate was found at 30 days with persistence at 90 days, which is also observed in the implantation area of the control sample.

The presence of lymphocytes in the implantation sites is associated with recognition and cellular communication, especially with macrophages in the chronic phase. A predominantly lymphocytic cell infiltrate was found at 30 days with persistence at 90 days, which is also observed in the implantation area of the control sample [89].

Very interestingly, samples F3 and F4 showed slightly different behavior from the other nanocomposites. A lymphoplasmacytic infiltrate was observed at 30 days, which is replaced at 90 days by a more lymphocytic-type infiltrate with the presence of some histiocytic-type cells. The cause of the very different behavior of samples F3 and F4 is possibly due to their composition that supports high non-covalent interactions between PVA/CS/ox-CNO (hydrogen bonds and van der Waals interactions) and higher crystalline degree. It is usually accepted that the degree of crystallinity influences the hydrolysis rate for polysaccharides (i.e., cellulose, starch, chitosan) [85]. The amorphous regions for a polysaccharide such as cellulose will suffer first enzymatic hydrolysis because water can quickly rise those regions, make hydrogen bonds with hydroxyl groups, and facilitate enzyme activity [85,90].

On the other hand, chitosan can be degraded by lysozymes, chitinase, and chitosanase, producing oligomers that activate the wound healing processes [88,91,92]. Thus, increasing the crystallinity will decrease the rate of enzymatic hydrolysis of the nanocomposite for F3 and F4 compared to F1 and F2, which finally affects the reabsorption rates and will explain the higher stability. The histological study of this area of implantation indicates that the fragments that persist after 90 days are surrounded by a layer of histiocytes, phagocytic cells that control the presence of this material, which despite being biocompatible, is considered a foreign body. The presence of histiocytes associated with biomaterials has already been reported for skin injections of polylactic acid and other materials [93,94].

## 3. Materials and Methods

### 3.1. Materials

The *p*-CNO (pristine carbon nano onions) synthesis followed the previous method reported [40]. The synthesis of ox-CNO was obtained by dispersing 100 mg of *p*-CNO in fuming sulfuric acid (H_2_SO_4_), nitric acid (HNO_3_), and sodium hydroxide (NaOH) (Merck, Burlington, MA, USA). Chitosan (*Mv* 144,000, Ubbelohde 0C viscometer, *K* and *a* constants in acetic acid 0.3 M + sodium acetate 0.2 M at 25 °C are 0.074 mL/g and 0.76, respectively) [95]. The molar mass was calculated using the Mark-Houwink-Sakurada equation (Equation (1)):

(1) $$\left[\eta \right]=K{\left(Mv\right)}^{a}$$

The CS deacetylation degree was 89–90% measured by ^1^H-NMR (using a BRUKER AVANCE II spectrometer with 400 MHz of frequency at a temperature of 300 K, (Bruker, Berlin, Germany) and elemental analysis (Thermo Electron Flash EA 1112, Thermo Fischer, Waltham, MA, USA). The sample was dissolved in D_2_O with two drops of trifluoroacetic acid as solvent and 3-(trimethylsilyl)propionic acid-d_4_ as reference salt. The PVA used for the nanofiber’s synthesis had a hydrolysis degree of 87–89% and a molar mass of 93,000 g/mol (Sigma-Aldrich, Palo Alto, CA, USA). Glacial acetic acid used for the preparation of CS solutions was provided by Merck (Burlington, MA, USA).

### 3.2. Methods

#### 3.2.1. Synthesis of Carbon Nano-Onions and Oxidized Carbon Nano-Onions

**Scheme 1:**
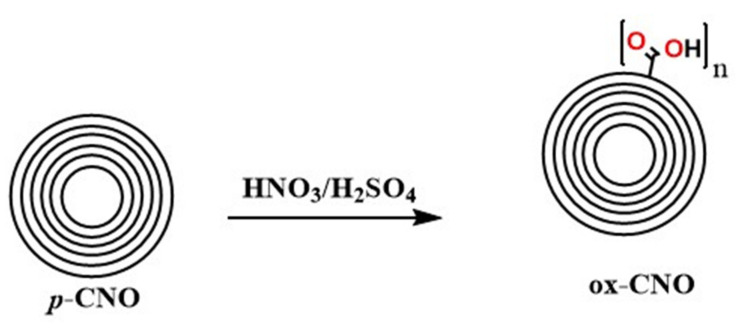
The oxidation of pristine carbon nano-onions (*p*-CNOs).

The oxidized carbon nano-onions (ox-CNO) were prepared through the rough oxidation of *p*-CNO (Scheme 1), as reported before [40].

#### 3.2.2. Preparation of Electrospun CS/PVA/ox-CNO Nanocomposites

The scaffold synthesis followed the electrospinning methodology under a vertical configuration. Initially, the mixture CS/PVA/ox-CNO was introduced into a 10 mL syringe coupled through a polypropylene hose to the stainless-steel electrospinning nozzle. Subsequently, different voltages were applied depending on the composition of the mixture employed (Table 3) with a high voltage source Model E30 (Gamma High Voltage Research INC., Ormond Beach, FL, USA), achieving a positive polarization of the solution. The latter is entrained utilizing a Braintree Scientific INC pump (Braintree, MA, USA), keeping the flow constant (Table 3) depending on the formulation used. The fibers were obtained by the attraction of the solution towards the collector, which is negatively charged about 15 cm from the surface (tip-to-collector distance: TCD) due to the solution positive polarization and the electric field generated by the electric field source. These fibers were collected in a Teflon mold covered by aluminum foil.

For the preparation of electrospun fibers, several formulations were prepared according to the methodologies implemented by our research group [33] (Table 4). Electrospun solutions were prepared mixing stock solutions of PVA 8% (*w/v*), and CS 3% (*w/v*), in acetic acid 2% (*v/v*). After that, we dispersed ox-CNO in 2% acetic acid (10 mg/mL) using an ultrasonic bath (Branson, Madrid, Spain) for one hour. Finally, all the components were mixed vigorously.

#### 3.2.3. Electrospun CS/PVA/ox-CNO Nanofibers Characterization

The functional groups of the fibers were characterized by Fourier-transform with a diamond tip (500–4000 cm^−1^ in transmittance mode) using a spectrophotometer FT-IR-8400 (Shimadzu, Kyoto, Japan).

##### X-ray Diffraction (DRX)

The X-ray spectrum (2θ between 5° and 50°) was obtained with a PANalytical X’Pert PRO diffractometer (Malvern PANalytical, Jarman Way, Royston, UK). Cu Kα1 radiation (1.540598 Å) and Kα2 (1.544426 Å). Electron accelerator voltage of 45 kV. Electron-generating current of 40 mA. Optical grid of incident beam 1°, and a diffracted beam grid of 9.1 mm.

The crystallinity percentage (*X_C_* %) was calculated using Equation (2):

(2) $$X_{C}\left(\%\right)=\left(\frac{A_{C}}{A_{T}}\right)\times 100$$

*A_C_* is the area under the peaks representing the crystalline region and *A_T_* total area of the crystalline and amorphous region [96].

##### Scanning Electron Microscopy (SEM)

The scaffold’s morphology was analyzed at 20 kV on secondary backscattered electrons using a scanning electron microscope (SEM) (JEOL JSM-6490LA, Musashino, Tokyo, Japan).

##### Thermal Studies

The scaffold’s thermal degradation (until 1000 °C, heating rate of 10 °C/min, air atmosphere, flow rate 80 mL/min) was analyzed on a TA Instruments TGA Q50 V20.13 Build 39 (TA instrument, New Castle, DE, USA). The glass transition temperature (Tg) and melting points (Tm) were obtained from the first heating cycle of the differential scanning calorimetry technique with a DSC2A-00181 TA instrument (New Castle, DE, USA) from the midpoint of the inflection tangent (heating at 10 °C/min).

##### Biomodels Tests In Vivo

The ISO 10996 (UNE 10996) standard guides the performance of dorsal subcutaneous implantations in adult mice, rats, guinea pigs, or rabbits to study biocompatibility in vivo. The standard also determines a minimum number of three biomodels to obtain reliable results and the use of a sufficient number of implantation sites (up to 10).

In this investigation, six Wistar rats were used, male four months of age and an average weight of 380 g. The biomodels were sedated by intramuscular application of a solution of Ketamine 30 mg/Kg (Holliday Scott S.A., Buenos Aires, Argentina) and Xilacina 70 mg/Kg (Xilaxyn-Virbac, Bogota, Colombia). Each biomodels underwent six surgical preparations 1 cm wide by 5 cm deep in dorsal subdermal areas.

In the surgical preparations, nanocomposite films with a dimension of 10 mm long by 5 mm wide corresponding to each of the five samples (F1–F4) were inserted, and in the sixth preparation, a Biomec porcine collagen membrane was inserted as a control (3Biomat, Bogota, Colombia).

After implantation periods of 30 and 90 days, euthanasia was performed with intraperitoneal sodium pentobarbital 100–150 mg/kg (Euthanex-INVET, Medellín, Colombia). The samples were recovered, and the fixation processes were carried out with formalin buffeted for 48 h. Dehydration of samples was performed with ascending alcohol (70%, 80%, 95%, and 100%), diaphanization with xylol, and infiltration with paraffin using the Autotechnicon Tissue Processor™ (Leica Microsystems, Mannheim, Germany) and immersed in paraffin blocks using the Thermo Scientific™ Histoplast Paraffin™ kit.

The samples were finally cut to 5 µm with the Leica microtome ((Leica Microsystems, Mannheim, Germany), staining with Hematoxylin–Eosin was performed, and the images were analyzed with an optical microscope with a camera included and equipped with the Leica suite application. (Leica Microsystems, Mannheim, Germany).

The bio-models were supplied and hosted by the Intermediate Laboratory for Preclinical Research and Murine Bioterium of the Universidad del Valle in Cali—Colombia. The Ethics Committee supervised the project with Biomedical experimentation animals of the same university through resolution CEAS 012-019. The samples studied for histological analysis used the hematoxylin and eosin (H–E) technique [38,40].

## 4. Conclusions

The synthesis of CS/PVA/ox-CNO nanofibers was confirmed using different characterization techniques. FT-IR spectra of nanofibers demonstrated the partial compatibility of the components through chemical interactions and hydrogen bonds. We found that the morphology and crystallinity of the nanofibers were influenced by parameters such as the properties of the solution (concentration, viscosity, and surface tension) and CS/PVA ratios. Comparing the XRD and DSC data in the nanofibers, we concluded that the electrospinning affected the crystallinity compared presumably by rapid solvent evaporation, elongation, and solidification before crystal formation. The nanocomposites F1–F4 showed stability during the implantation after 90 days. Samples F3 and F4 show less reabsorption due to the increasing crystallinity rate, decreasing the rate of enzymatic hydrolysis of the nanocomposite for F3 and F4, as compared for F1 and F2, which finally affects the reabsorption rates, and it will explain the higher stability. All samples presented a lymphocytic infiltrate component at 30 days that decreased to mild at 90 days, including the porcine collagen control sample, which is considered a healthy reabsorption process. Sample F3 generated a more significant inflammatory response characterized by a lymph-plasmacytic inflammatory infiltrate at 30 days, replaced by a mainly lymphocytic infiltrate with some histiocytes at 90 days, without considering an aggressive reaction immune system. The plausible reason for this behavior might be that F3 was more crystalline, which directly influences the rate of enzymatic hydrolysis. Additionally, the nanocomposites are more resistant to degradation, which led the organism to try to control it with the second line of cells such as plasma cells and lymphocytes, unlike the other samples that, as they are degraded/reabsorbed, there is an infiltrate by lymphocytes that decreases notably by the third month in all cases. All the above results demonstrate the importance of incorporating ox-CNO to reinforce CS-PVA nanofibers without affecting the biocompatibility process while resisting rapid growth enzymatic hydrolysis in body conditions. All these results demonstrate that nanofibers prepared here are valuable for tissue engineering and biomedicine applications.

## Figures and Tables

**Table 1:** Crystallinity index from XRD (Xc), Tg, and endothermic peaks for the electrospun nanocomposites.

Formulation	Xc (%)	Tg (°C)	Tm (°C)
F1	0.86	93	192
F2	1.68	94	193
F3	8.17	92	195
F4	12.5	89	193

**Table 2:** Average diameters of the nanofibers CS/PVA/ox-CNO from the SEM micrographs.

Formulation *	Average Diameter (nm)	Standard Deviation (±nm)
F1	147.8	29.4
F2	120.7	11.9
F3	117.9	15.9
F4	102.1	12.9

* Formulations: F1 (CS:PVA:ox-CNO 8:92:0); F2 (CS:PVA:ox-CNO 8:91:1); F3 (CS:PVA:ox-CNO 3.5:95.5:1); F4 (CS:PVA:ox-CNO 5.4:92.6:2).

**Table 3:** Experimental conditions for the electrospinning procedure of the nanocomposites CS/PVA/ox-CNO.

Variable	F1	F2	F3	F4
TCD (cm)	20	15	15	15
Injection rate (mL/h)	0.13	0.6	0.6	0.85
Voltage (kV)	16	18.5	17	15

**Table 4:** Formulation composition of nanocomposites CS/PVA/ox-CNO.

Component	F1	F2	F3	F4
CS (%)	8	8	3.5	5.4
PVA (%)	92	91	95.5	92.6
ox-CNO (%)	0	1	1	2.0

## Data Availability

The data presented in this study are available on request from the corresponding author.

## Supplementary Materials

The following are available online, Figure S1. TGA curves of the components CS (A), PVA (B), *p*-CNO vs. ox-CNO (C), and differential of *p*-CNO vs. ox-CNO (D). Figure S2. SEM images including diameters of electrospun CS/PVA/ox-CNO composite nanofibrous membranes Formulations: F1 (CS:PVA:ox-CNO 8:92:0); F2 (CS:PVA:ox-CNO 8:91:1); F3 (CS:PVA:ox-CNO 3.5:95.5:1); F4 (CS:PVA:ox-CNO 5.4:92.6:2).

## Sample Availability

Samples of the compounds are not available from the authors.
