# Supplementary material for: Biocompatibility Study of Electrospun Nanocomposite Membranes Based on Chitosan/Polyvinyl Alcohol/Oxidized Carbon Nano-Onions
Source: Molecules. 2021 Aug 6;26(16):4753. doi: 10.3390/molecules26164753 (PMC8400231; doi:10.3390/molecules26164753)
Supplement: Supplementary file 1 [file molecules-26-04753-s001.zip › molecules-1304430-supplementary.pdf]

Supplementary Material

Biocompatibility Study of Electrospun Nanocomposite Membranes Based on Chitosan/Polyvinyl Alcohol/Oxidized Carbon Nano-Onions

Jorge Iván Castro [1], Manuel N. Chaur [1], Carlos Humberto Valencia Llano [2], Mayra Eliana Valencia Zapata [3], José Herminsul Mina Hernandez [3] and Carlos David Grande-Tovar [4,*]

[1] Grupo de Investigación SIMERQO, Departamento de Química, Universidad del Valle, Calle 13 No. 100-00, 76001 Cali, Colombia; jorge.castro@correounivalle.edu.co (J.I.C.); manuel.chaur@correounivalle.edu.co (M.N.C.)

[2] Grupo Biomateriales Dentales, Escuela de Odontología, Universidad del Valle, Calle 4B No. 36-00, 76001 Cali, Colombia; carlos.humberto.valencia@correounivalle.edu.co

[3] Grupo de Materiales Compuestos, Escuela de Ingeniería de Materiales, Facultad de Ingeniería, Universidad del Valle, Calle 13 No. 100-00, 760032 Santiago de Cali, Colombia; valencia.mayra@correounivalle.edu.co (M.E.V.Z.); jose.mina@correounivalle.edu.co (J.H.M.H.)

[4] Grupo de Investigación de Fotoquímica y Fotobiología, Facultad de Ciencias, Universidad del Atlántico, Carrera 30 Número 8-49, 081008 Puerto Colombia, Colombia

* Correspondence: carlosgrande@mail.uniatlantico.edu.co; Tel.: +57-53-599-484

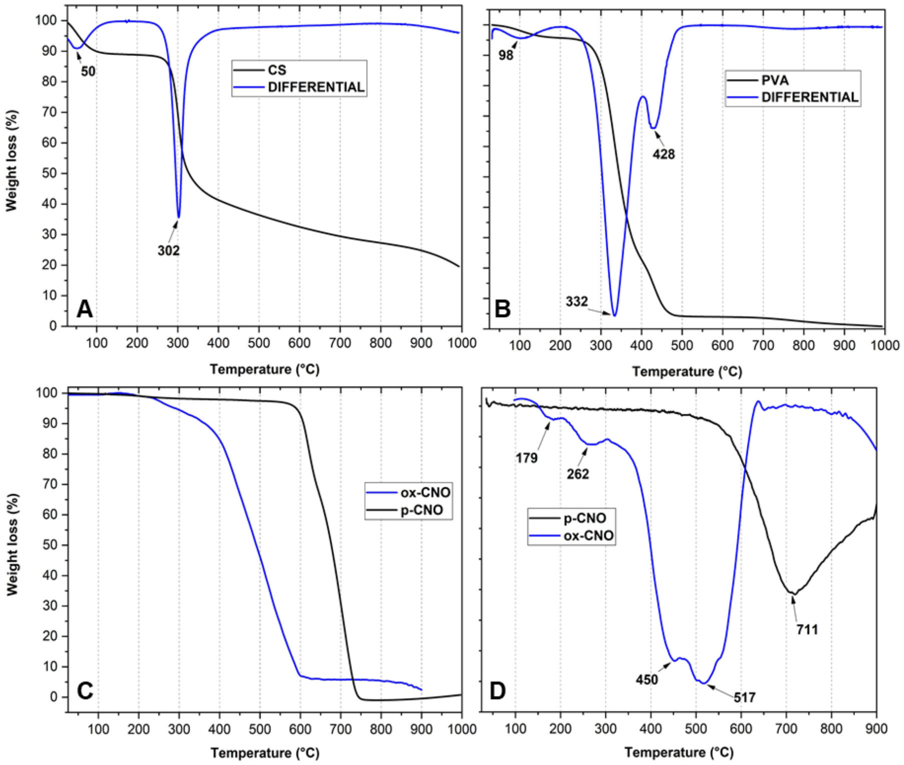

Figure S1. TGA curves of the components CS (**A**), PVA (**B**), *p*-CNO vs. ox-CNO (**C**), and differential of *p*-CNO vs. ox-CNO (**D**).

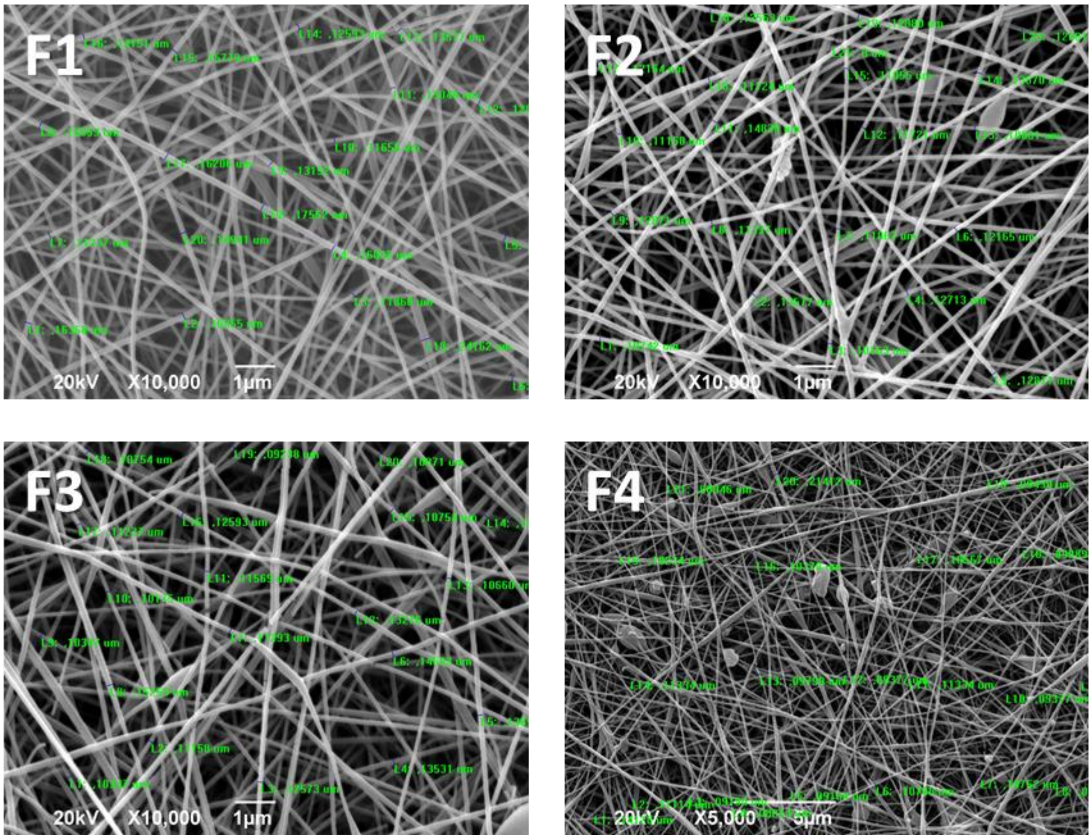

Figure S2. SEM images including diameters of electrospun CS/PVA/ox-CNO composite nanofibrous membranes formulations: **F1** (CS:PVA:ox-CNO 8:92:0); **F2** (CS:PVA:ox-CNO 8:91:1); **F3** (CS:PVA:ox-CNO 3.5:95.5:1); **F4** (CS:PVA:ox-CNO 5.4:92.6:2). For all experiments, the voltage used was 20kV.
